# Electrophysiological Effects of Transcranial Direct Current Stimulation on Neural Activity in the Rat Motor Cortex

**DOI:** 10.3389/fnins.2020.00495

**Published:** 2020-06-30

**Authors:** Tomoko Tanaka, Yoshikazu Isomura, Kazuto Kobayashi, Takashi Hanakawa, Satoshi Tanaka, Manabu Honda

**Affiliations:** ^1^Department of Brain Development and Neural Regeneration, Tokyo Metropolitan Institute of Medical Science, Setagaya, Japan; ^2^Department of Information Medicine, National Institute of Neuroscience, National Centre of Neurology and Psychiatry, Kodaira, Japan; ^3^Physiology and Cell Biology, Graduate School of Medical and Dental Sciences, Tokyo Medical and Dental University, Bunkyo, Japan; ^4^Department of Molecular Genetics, Institute of Biomedical Sciences, Fukushima Medical University School of Medicine, Fukushima, Japan; ^5^Department of Advanced Neuroimaging, Integrative Brain Imaging Centre, National Centre of Neurology and Psychiatry, Kodaira, Japan; ^6^Laboratory of Psychology, Hamamatsu University School of Medicine, Hamamatsu, Japan

**Keywords:** tDCS, motor cortex, neuronal activity, single unit, multiunit activity

## Abstract

Transcranial direct current stimulation (tDCS) is a non-invasive technique that modulates the neuronal membrane potential. We have previously documented a sustainable increase in extracellular dopamine levels in the rat striatum of cathodal tDCS, suggesting that cathodal tDCS enhances the neuronal excitability of the cortex. In the present study, we investigated changes in neuronal activity in the cerebral cortex induced by tDCS at the point beneath the stimulus electrode in anesthetized rats *in vivo*. Multiunit recordings were performed to examine changes in neuronal activity before and after the application of tDCS. In the cathodal tDCS group, multiunit activity (indicating the collective firing rate of recorded neuronal populations) increased in the cerebral cortex. Both anodal and cathodal tDCS increased the firing rate of isolated single units in the cerebral cortex. Significant differences in activity were observed immediately following stimulation and persisted for more than an hour after stimulation. The primary finding of this study was that both anodal and cathodal tDCS increased *in vivo* neuronal activity in the rat cerebral cortex underneath the stimulus electrode.

## Introduction

Transcranial direct current stimulation (tDCS) modifies motor and cognitive functioning, presumably by altering the neuronal activity of the stimulated sites (Tanaka et al., 2009; Bachmann et al., 2010; Groppa et al., 2010; Fujimoto et al., 2014). Therefore, tDCS has the potential for use as an adjuvant strategy in the rehabilitation of motor and cognitive deficits caused by neurological disorders (Hummel et al., 2005; Doruk et al., 2014; Penolazzi et al., 2014; Saeys et al., 2014; Sakrajai et al., 2014). The effects of tDCS depend on the polarity of stimulation. Anodal tDCS induces physiological responses, such as enhancement of motor evoked potential (MEP) size or visual evoked potential (VEP) size, by depolarizing the resting membrane potential (Nitsche and Paulus, 2000; Cambiaghi et al., 2010, 2011). The increase in excitability of a stimulated region is associated with improved brain function, such as working memory and muscular control (Ohn et al., 2008; Cerruti and Schlaug, 2009; Tanaka et al., 2009). Conversely, cathodal tDCS induces physiological responses such as decreased MEP size or VEP size by hyperpolarizing the resting membrane potential (Nitsche and Paulus, 2000; Cambiaghi et al., 2010, 2011). The decrease in excitability observed in a stimulated region is associated with effects on brain function, such as deficits in motor learning and muscular control (Stagg et al., 2011; Young et al., 2013). Based on these results, it is thought that anodal and cathodal tDCS facilitate and suppress brain functions, respectively (Lang et al., 2005; Wassermann and Grafman, 2005). Such polarity-dependent effects of tDCS have been verified in clinical studies (Luft et al., 2017). However, several studies have reported asymmetrical effects of anodal versus cathodal tDCS on cognitive and motor functions (Bienenstock et al., 1982; Antal et al., 2007, 2014; Bradnam et al., 2010; Monte-Silva et al., 2010; Fricke et al., 2011; Bolzoni et al., 2013; Amadi et al., 2014; Falcone et al., 2018); these effects are potentially caused by motor-cognitive interference during stimulation, intersubject variation, and the stability of individual subjects.

We have previously reported that cathodal tDCS for 10 min induced a significant sustainable increase in extracellular dopamine levels in the rat striatum. This phenomenon indicates that cathodal tDCS may induce an acute or sustainable increase of neuronal activity in the cortex, in contradiction to long-standing hypotheses (Tanaka et al., 2013). Many studies of the effects of DCS on neuronal excitability involved *in vitro* investigations or direct current applied to the dendrites of recorded cells *in vivo* (Bindman et al., 1964; Purpura and McMurtry, 1965). These effects may differ from those arising from transcranial stimulation via the skin. Indeed, the cell-specific effects of tDCS differ from those of cell populations in specific regions. To define the mechanisms of tDCS, it is necessary to clarify the outputs from cell populations in specific regions.

Thus, we examined the mechanisms of tDCS in stimulated regions by investigating the tDCS-induced changes in multiunit activity and in the firing rates of single units in the rat cerebral cortex beneath the stimulus electrode *in vivo*, to clarify more accurately the effect of tDCS on biological responsiveness. Because tDCS exerts acute and continuous effects via different pathways (Nitsche and Paulus, 2000; Hummel et al., 2005; Power et al., 2006), we conducted an electrophysiological recording up to 120 min after stimulation to investigate the effects of both.

## Materials and Methods

### Ethical Approval

The experimental protocol was approved by the Animal Care and Use Committee of the National Institute of Neuroscience (National Center of Neurology and Psychiatry, Tokyo, Japan). The experiments were conducted in accordance with the “Official Notification on Animal Experiments” (National Institute of Neuroscience, National Center of Neurology and Psychiatry notification no. 2010004, received 2010). Every effort was made to minimize the number of animals used in the experiments and their suffering.

### Animals

Male 9-week-old Sprague Dawley rats (CLEA Japan, Inc., Tokyo, Japan) were housed at a temperature of 23 ± 1°C with a 12-h light/dark cycle (lights on 08:00–20:00). Food and water were available *ad libitum*. For the electrophysiological experiments, 32 rats were used. The rats (*n* = 32) were divided into three groups: the sham group (*n* = 11), cathodal tDCS group (*n* = 10), and anodal tDCS group (*n* = 11).

### tDCS

**FIGURE 1:**
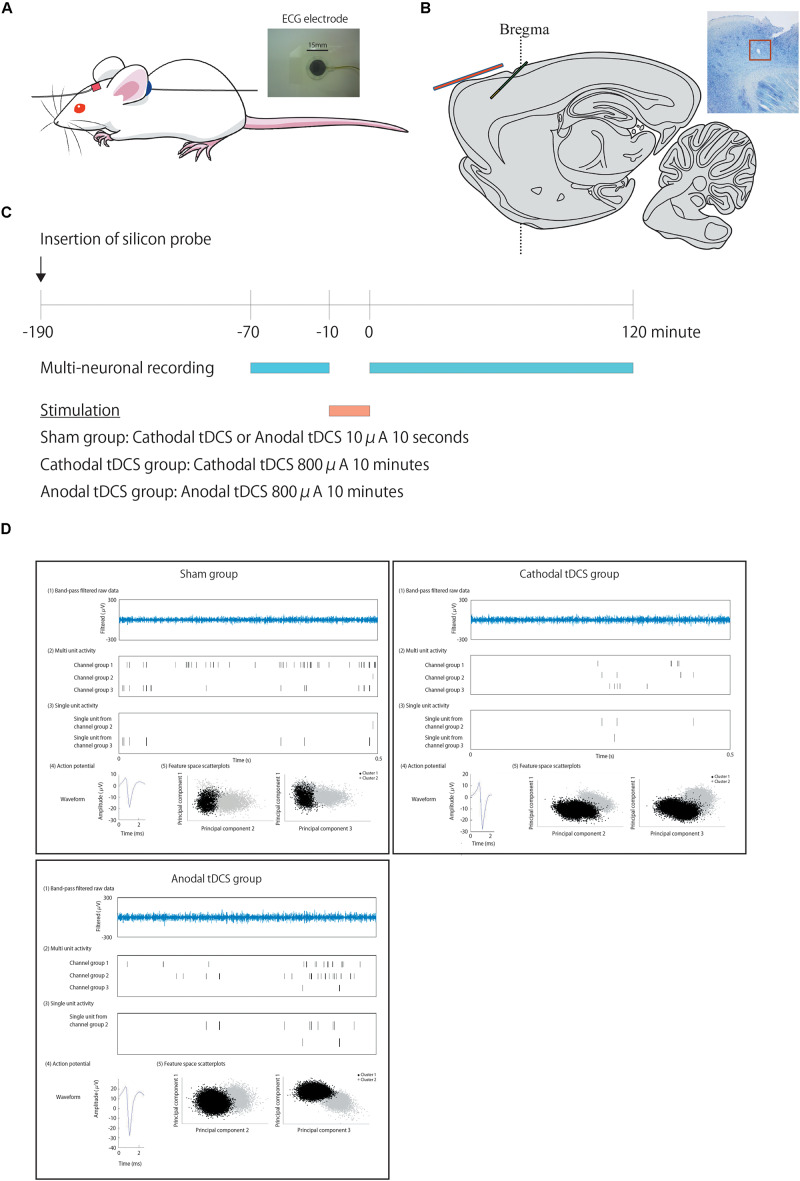
Positions of the transcranial direct current stimulation (tDCS) and recording electrodes, experimental schedule, and typical data traces. **(A)** We used commercial ECG electrodes containing a tacky gel on the surface of the electrode and DIN connector (a photograph). One electrode of the stimulator, a 5 × 5-mm square, is fixed with surgical tape to the skin of the rat scalp about 1 mm in the frontal lobe to the recoding electrode probe hole (in red) **(A)**, while a second electrode, without any size reduction, was placed on the neck (in blue). **(B)** A one-shank, 16-channel silicon probe is inserted as a recording electrode through a hole in the skull into the cortex at an angle of 45° immediately beneath the tDCS electrode in green. This illustration shows the positional relationship of the recording electrode in green to the electrode of stimulator in red. **(C)** After insertion of the probe and confirmation of stability, multiunit recording commences. After 60 min of recording baseline activity, cathodal or anodal tDCS is applied over the cortex. Following the offset of stimulation, at time 0, multiunit recording is performed for an additional 120 min. **(D)** Multiunit recording data were processed to isolate spike events with the automatic spike-sorting software EToS featuring wavelet transform and robust variational Bayes. A representative data of multiunit recording in each group before stimulation is shown. The steps to calculate these signals from the raw data are as follows. To calculate the firing rate of single units, raw data were automatically processed to isolate spike events. [The first of five examples: “Band-pass-filtered row data” in plots are representative examples of band-pass-filtered row data. The second of five examples: “Multiunit activity” in plots are representative examples of multiunit activity (pre-isolated unit)]. Spike channel groups two and three without channel group one, which contained a false-negative spike and synchronized spikes that were detected as a single spike, are then refined semiautomatically into single-unit contributions (The third of five examples: “Single-unit activity” in plots is a representative example of isolated single units from each channel group. The fourth of five examples: “Action potential” in plots is a representative example of action potentials from each channel group. The fifth of five examples: “Feature space scatterplots” in plots are representative examples of feature space scatterplots using three of the nine feature dimensions).

The experimental tDCS setup was similar to that reported by Tanaka et al. (2013). We used commercial ECG electrodes containing a tacky gel on the surface of the electrode and DIN connector (photograph in Figure 1A: Biorode; Nihon Kohden, Corp., Tokyo, Japan). One electrode of the stimulator, a 5 × 5-mm square, is fixed with surgical tape to the skin of the rat scalp about 1 mm in the frontal lobe to the recording electrode probe hole (in red) (Figure 1A), while a second electrode, without any size reduction, was placed on the neck (in blue).

Cathodal or anodal tDCS was applied continuously for 10 min in the experimental groups with a current intensity of 800 μA from the electrode on the scalp using a DC stimulator (STG1002; Multi Channel Systems, Germany). Cathodal or anodal tDCS was applied for 10 s to the sham group with a current intensity of 10 μA from the electrode on the scalp. All rats were stimulated once during experiments. The safety limit of the stimulator was 120 V; current intensity of 800 μA, corresponding to a current density of 32.0 A/m^2^ and charge density of 19.2 kC/m^2^ in the present setting, was used to maximize the effects of tDCS within the safety limits reported in previous rat tDCS studies (Liebetanz et al., 2009; Jackson et al., 2017). We ensured that no abnormal findings regarding cellular morphology were observed in the cortex below the scalp electrode of rats by the stimulation (Tanaka et al., 2013).

### Electrophysiological Recordings

After ≥3 days of habituation to the animal colony, all rats were anesthetized intraperitoneally (i.p.) with a single shot of urethane (1 g/kg of body weight) and placed in a stereotaxic apparatus. Part of the skull was exposed, and a small hole was made using a dental drill. A barrier made of dental cement was placed to avoid the effects of direct current around the skull. Following surgery, a one-shank, 16-channel silicon probe, which contained two sets of tetrode-like electrodes (at the tip and 800 μm above it) for measuring unit activity and eight electrodes for measuring local field potentials (LFP8 + TetrodeSD; NeuroNexus Technologies), was inserted into the rat cerebral cortex at an angle of 45° (insert onset position: 0.0 mm anterior, 3.5 mm lateral, and −0.5 mm ventral to bregma; the tip of the electrode position was estimated to be located at 2.3 mm anterior, 3.5 mm lateral, and 1.8 mm ventral to bregma) immediately beneath the tDCS stimulus electrode through a hole in the skull (Figure 1B). After confirmation of stability, we performed multiunit recording to collect baseline data (sampling rate, 20 kHz; final gain, 2000; original band-pass filter, 0.5–10 kHz). After 60 min, cathodal or anodal tDCS was applied over the cortex, including the cerebral cortex. Following application of tDCS, multiunit recording was performed for a further 120 min or more after offset of stimulation. The offset of stimulation time was used as the zero point on the time axis. See Figure 1C.

### Spike Activity Analysis

Data points were 10 min apart on the horizontal axis. Multiunit recording data were processed to isolate spike events with the automatic spike-sorting software EToS featuring wavelet transform and robust variational Bayes (Takekawa et al., 2012). Spike channel groups were combined/divided/discarded manually to define single-unit contributions by nine feature dimensions using the manual clustering software Klusters and NeuroScope (Hazan et al., 2006) (sham group, *n* = 127; cathodal tDCS group, *n* = 135; anodal tDCS group, *n* = 122). The relationship between spike activity and the effects of tDCS was analyzed using MATLAB (MathWorks). The ongoing and baseline spike rates, spike duration, spike width, and the onset and peak of phasic activation were all defined in the manner described previously (Isomura et al., 2013). Single units were segregated according to the change in firing rate from baseline, as assessed by an unpaired *t*-test or Wilcoxon signed-rank test based on a normality test (units with increased, decreased, or unchanged firing rates following stimulation relative to the rates at baseline).

### Histological Observations

To confirm the insertion positions of the recording electrodes after completion of experiments, direct current was applied for 5 s with a current intensity of 50 μA via the recording electrode to electrolytically mark the site after recording. The rats were then deeply anesthetized with sodium pentobarbital (50 mg/kg of body weight, i.p.) and perfused through the heart sequentially with 1 × phosphate-buffered saline followed by 10% formalin in neutral buffer solution. The rat brains were postfixed, cryoprotected in sucrose at 4°C, and sliced into 30-μm-thick sagittal sections through the right side of the cortex using a cryostat. The sections were then thaw-mounted on 3-aminopropyltriethoxysilane-coated slides and stained with cresyl violet using standard procedures.

### Statistical Analysis

The multiunit recording data are expressed as the mean ± standard error. Statistical analysis was performed using R software^1^ and JMP (SAS Institute Inc.). For multiunit activity, the statistical significance of the differences between groups was assessed by a repeated-measures analysis of variance (ANOVA) with time (TIME) as a within-subject factor and group (GROUP) as a between-subjects factor. This was followed by a *post hoc* Holm test. To investigate whether the time effect differed among groups, we confirmed the TIME × GROUP interaction. Differences with *p*-values of <0.05 were considered statistically significant. To investigate the homogeneity of the firing rate of single units during the 60 min before interventions, the data in each group were compared using the chi-squared test. Next, in each single unit, we calculated the average of firing rate (spikes per minute) over 10 min prior to stimulation and the average of firing rate per minute during 120 min following stimulation. We compared the proportion of average firing rates prestimulation and poststimulation by group using the chi-squared test. Single units were segregated according to the change in firing rate from baseline (unpaired *t*-test or Wilcoxon signed-rank test based on a normality test; *p* < 0.01; the resulting groups were units with increased, decreased, or unchanged firing rates following stimulation relative to those at baseline). We calculated the number of units with increased, decreased, and unchanged firing rate at time points of 10-min intervals following stimulation. We performed a chi-squared test and Holm test for multiple comparisons between groups to compare the proportion of units at each time point using the number of units.

## Results

### Experimental Design

Figure 1 shows the positions of the tDCS electrodes and recording electrodes (Figures 1A,B), the experimental schedule (Figure 1C), and a representative example of analysis data (Figure 1D).

### tDCS-Induced Increases in Multiunit Activity

**FIGURE 2:**
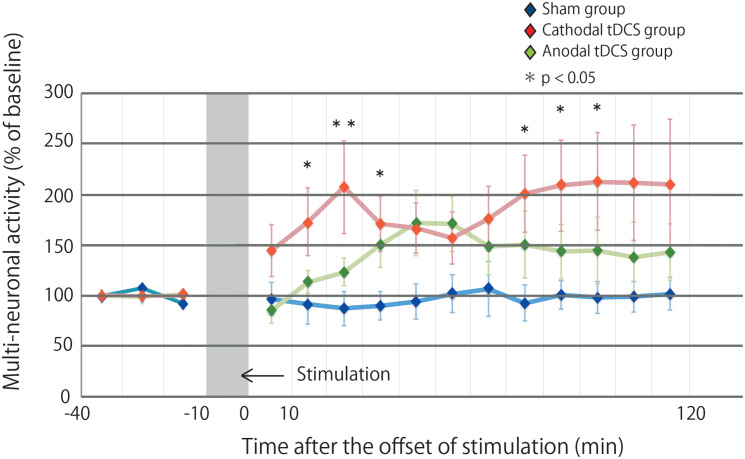
Multiunit activity increases in the stimulated cortex after tDCS. Time series data of multiunit activity are expressed as the percentage change from baseline attributable to tDCS. Group data are presented as the mean ± standard error of the mean. The increase in multiunit activity in the cathodal tDCS group is significantly greater than that in the sham group. Multiunit activity in the cathodal tDCS group is significantly increased compared with that in the sham group at several time points.

Figure 2 illustrates the effects of tDCS on multiunit activity, which indicates the collective firing rates of recorded neuronal populations. The multiunit activity in the sham group was not altered by stimulation. In contrast, the multiunit activity in the cathodal and anodal groups was increased by stimulation, and the effects were maintained for 120 min after stimulation. A repeated-measures ANOVA revealed a significant main effect of group on multiunit activity, whereby the effects of stimulation differed among the three groups [main effect of GROUP, *F*_(__2_,_29__)_ = 3.4569, *p* = 0.0450; main effect of TIME, *F*_(__14_,_406__)_ = 3.0361, *p* = 0.00002; interaction of GROUP × TIME, *F*_(__28_,_406__)_ = 1.5249, *p* = 0.0445].

*Post hoc* analysis indicated that the multiunit activity after stimulation in the cathodal tDCS group was significantly increased compared with that in the sham group (*p* = 0.0135). In the anodal tDCS group, the multiunit activity after stimulation did not significantly differ from that in the sham group (*p* = 0.2016). The multiunit activity after stimulation did not significantly differ between the cathodal and anodal tDCS groups (*p* = 0.1862). These results suggest that cathodal tDCS enhances neuronal activity in the cerebral cortex.

The multiple comparison of the cathodal tDCS group and the sham group at each time point indicated that the multiunit activity in the cathodal tDCS group was significantly increased compared with that in the sham group at several time points (10–20 min following stimulation: *p* = 0.0223; 20–30 min following stimulation: *p* = 0.0080; 30–40 min following stimulation: *p* = 0.0226; 70–80 min following stimulation: *p* = 0.0272; 80–90 min following stimulation: *p* = 0.0299; 90–100 min following stimulation: *p* = 0.0241). Differences were observed both immediately following stimulation and persisted for more than an hour following stimulation, indicating persistent cathodal effects on the neuronal firing rate. The multiple comparison of the anodal tDCS group and the sham group at each time point indicated that the multiunit activity in the anodal tDCS group did not significantly differ from that in the sham group at several time points.

### tDCS-Induced Increases in the Firing Rates of Single Units

**FIGURE 3:**
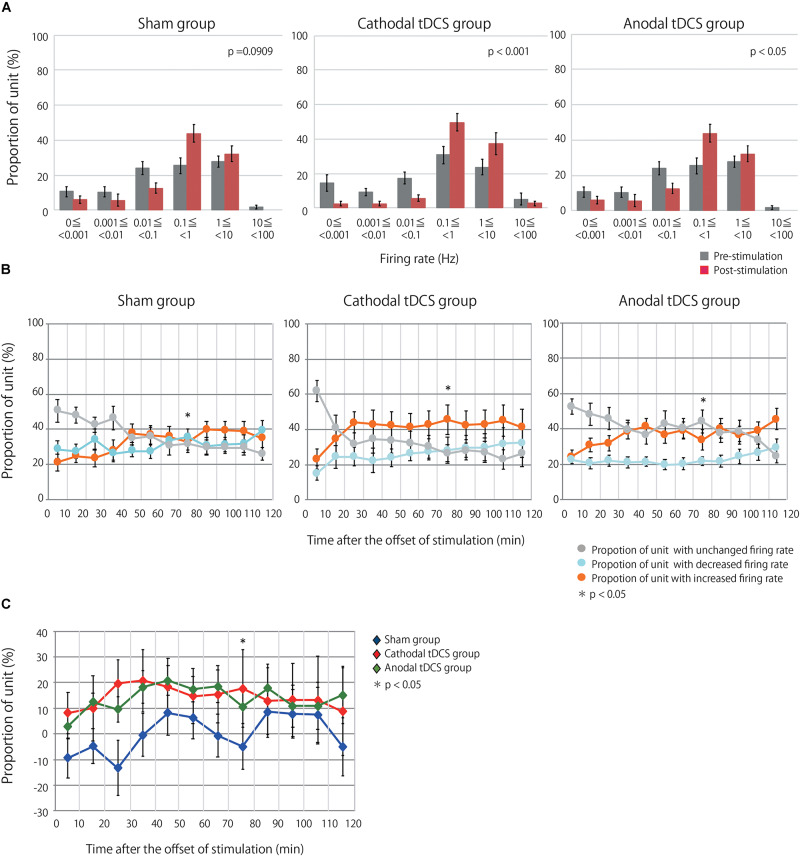
tDCS induced an increase in the firing rates of single units in the stimulated cerebral cortex. **(A)** Group data are the proportion of unit by firing rate during prestimulation and poststimulation. Each datum is presented as the mean ± standard error of the mean. The proportion of unit after stimulation in the anodal and cathodal tDCS groups significantly differed from that before stimulation. **(B)** The proportions of neurons with increased, decreased, or unchanged firing rates over three groups at time points of 10-min intervals following stimulation. Each datum is presented as the mean ± standard error of the mean. **(C)** The proportion of neurons with increased firing rates after tDCS minus the proportion with decreased firing rates. Each datum is presented as the mean ± standard error of the mean. The distribution of the proportions significantly differed among the three groups at 70–80 min following stimulation.

Multiunit activity represents the population-based behavior of recorded neurons but obscures the behavior of individual neurons (Martinez et al., 2009). Therefore, the multiunit activity was segregated into the firing contributions of single units, and we then investigated the effects of tDCS on individual neurons. We identified 127, 135, and 122 neurons in the sham, cathodal, and anodal tDCS groups, respectively. Figure 3A indicates the proportion of neurons by firing rate for both the average of the 10 min before stimulation and the average of the 120 min after stimulation. Though after stimulation the proportion of neurons in the sham group was little different from that before stimulation, the proportions of neurons after stimulation in the anodal and cathodal tDCS groups appear to shift the firing rate toward high frequency. A chi-squared test between prestimulation and poststimulation by group indicated that the proportion of neurons after stimulation in the anodal and cathodal tDCS groups significantly differed from that before stimulation (Figure 3A; sham group χ^2^ = 9.351, df = 5, *p* = 0.0909; cathodal tDCS group χ^2^ = 28.127, df = 5, *p* < 0.0001; anodal tDCS group χ^2^ = 16.496, df = 5, *p* = 0.0037).

Single units were then grouped according to the change in firing rate from baseline, as assessed by unpaired *t*-test or Wilcoxon signed-rank test based on a normality test. This resulted in three groups, namely, units with increased, decreased, or unchanged firing rates following stimulation relative to baseline. Figure 3B shows the proportions of units with increased, decreased, or unchanged firing rates in the three groups. A chi-squared test followed by a Holm test for multiple comparisons indicated that the distribution of the proportions significantly differed among the three groups at 70–80 min following stimulation: χ^2^ = 15.711, df = 4, *p* = 0.0408). Figure 3C shows the difference between the proportion of units with increased firing rates and the proportion of neurons with decreased firing rates. The results from the analysis of single-unit activity support those of multiunit recordings, suggesting that anodal and cathodal tDCS can increase neuronal activity following stimulation in the cerebral cortex.

## Discussion

The main finding of this study was that both anodal and cathodal tDCS can increase *in vivo* neuronal activity in the rat cerebral cortex beneath the stimulus electrode. It is thought that anodal and cathodal tDCS have opposing functions, but our results suggest that the effects of tDCS are not completely imitative of electrical polarity.

Here, we investigated changes in the multiunit activity and firing rates of single units in the rat cerebral cortex beneath the stimulus electrode induced by tDCS under *in vivo* conditions, which resemble natural physiological conditions. We demonstrated that both anodal and cathodal tDCS can induce an increase in multiunit activity, which is representative of the neuronal activity at the population level. Although the persistent effects of anodal and cathodal tDCS began to develop at different time points, anodal and cathodal tDCS could induce persistent increases in neuronal excitability after stimulation. To define the effects at the individual neuron level, we investigated the effect of tDCS on the firing rates of single units. Although multiunit activity was typically increased by tDCS and the average firing rate of single units was increased by tDCS, single units exhibited asymmetric responses, with both increased and decreased firing rates observed. These effects are likely related to increased neuronal excitability induced by anodal and cathodal tDCS. Both anodal and cathodal tDCS had persistent effects on single-unit activity.

Physiological and behavioral studies on tDCS have been usually conducted under the hypothesis that anodal tDCS increases neuronal activity while cathodal tDCS decreases it. Early reports supporting this hypothesis investigated the effects of tDCS on neuronal excitability *in vitro* or applied direct current to a dendrite of a recorded cell *in vivo* (Bindman et al., 1964; Purpura and McMurtry, 1965). However, many studies have found asymmetrical effects of anodal and cathodal tDCS, which is contradictory to this long-standing hypothesis (Marquez-Ruiz et al., 2012; Ranieri et al., 2012; Amadi et al., 2014; Fertonani and Miniussi, 2016; Lafon et al., 2017). Furthermore, it has been reported that the dendrites and soma of a single neuron have differential responses to tDCS and that apical and basal dendrites also exhibit differential responses to tDCS (Bikson et al., 2004; Kronberg et al., 2017; Lafon et al., 2017). Kronberg et al. (2019) also demonstrated that tDCS has both symmetrical and asymmetrical effects based on somatic spiking and that the effect of tDCS is influenced by exogenous input *in vitro*. We also need to consider the effects within neuronal networks and cell assemblies. It has been demonstrated that anodal and cathodal tDCS of the motor cortex modulate resting brain dynamics in the frontoparietal motor network in the same direction (Venkatakrishnan et al., 2011). tDCS triggers a profound and sustainable reorganization of network connectivity and leads to the formation of cell assemblies according to computational modeling (Lu et al., 2019). In fact, tDCS delivered to the motor cortex mainly increases a functional connectivity between the ventroposterolateral (VPL), sensory nucleus of the thalamus and sensory networks, while tDCS to the dorsolateral prefrontal cortex increases functional connectivity between VPL and sensory networks and between the medial dorsal (MD) affective nucleus of the thalamus and affective networks (Sankarasubramanian et al., 2017). Parts of effects on brain region such as nucleus of the thalamus, other than the stimulated region, may also show asymmetry (Marquez-Ruiz et al., 2012). Recently, it has been demonstrated that not only anodal but also cathodal tDCS inhibit dorsal raphe nuclei 5-HT neurons through the activation of neurons in the prelimbic cortex, which is a stimulated region in mice (Cambiaghi et al., 2020). A meta-analysis showed that homogeneity/heterogeneity of tDCS effects depends on the target region. It has been indicated that cathodal inhibition is rarely observed when stimulating non-motor regions, suggesting that the lack of inhibitory cathodal effects may reflect compensatory processes in cognitive function (Jacobson et al., 2012). Although we focused on the motor cortex, we cannot allege that the stimulation remained within the motor cortex. The indirect, downstream effects of tDCS on non-motor regions should also be considered in the present study. Our previous results suggested that tDCS has a direct and/or indirect effect on the dopaminergic system in the rat striatum (Tanaka et al., 2013). These findings suggest that tDCS may alter cortical activity in both proximal and distal brain regions by modulating neurotransmitter activity. It has been demonstrated that cathodal tDCS reduced glutamatergic neuronal activity together with a correlated reduction in GABA (Stagg et al., 2009). Given this report, cathodal tDCS may not induce a state of simple excitability dominance or inhibitory dominance but rather an alteration of the balance of glutamatergic and GABAergic neurons, which results in the increase in neuronal activity. Thus, the current tDCS literature demonstrates three key findings: (1) differentially altered firing rates are observed following current injection into the soma versus dendrites of a single neuron; (2) responses to tDCS are not uniform across neurons and depend on the direction of the local current flow; and (3) changes in the proportions of units with increased, decreased, or unchanged firing rates induce changes in multiunit activity, leading to changes at the behavioral level.

It has been reported that cathodal direct current injection (DCS) enhances long-term potentiation (LTP) in apical dendrites, while anodal DCS enhances LTP in basal dendrites. Furthermore, both anodal and cathodal DCS reduce long-term depression (Ltd.) in apical dendrites in rat hippocampal slices (Kronberg et al., 2017). Notably, these synaptic plasticity changes are prolonged, and anodal and cathodal tDCS drive synaptic plasticity depending on specific factors. Our findings suggest that increased neuronal activity induced by anodal or cathodal tDCS in single units and neuronal populations may alter processes of synaptic plasticity, including LTP and Ltd. These results also suggest that anodal and cathodal tDCS induce sustained increases in the excitability of single units and in multiunit activity. tDCS exerts both acute and continuous effects (Nitsche and Paulus, 2000; Hummel et al., 2005; Power et al., 2006). The continuous effects are related to neuronal plasticity, including *N*-methyl-D-aspartate (NMDA) receptors (Nitsche et al., 2004a, b; Ardolino et al., 2005; Monai et al., 2016; Kronberg et al., 2017); accordingly, tDCS may regulate LTP and Ltd. via these receptors.

It is commonly thought that alterations in synaptic plasticity alter the responses to various external or internal factors. Recently, it was reported that the effects of tDCS depend on activity levels at resting or active states and on the task performed during tDCS stimulation (Antal et al., 2007; Bradnam et al., 2010; Monte-Silva et al., 2010; Fricke et al., 2011; Gill et al., 2015). Furthermore, the position and polarity of the electrode as well as its electrical resistance influence the effects of tDCS as described above (Im et al., 2008; Batsikadze et al., 2013; Bikson et al., 2013). There is growing recognition that the effects of tDCS are not solely dependent on these factors. In the future, activity-based differences in the effects of tDCS should be clarified.

There are several limitations of the present study. The first limitation is that general anesthesia affects brain metabolism, neuronal activity, and responses to sensory stimuli and pain (Friedberg et al., 1999). Conversely, responses to sensory stimuli and pain influence anesthesia state and affect firing rate. Although it is highly unlikely that a 10-min stimulation induced persisting somatosensory perception or pain response, it is quite possible that somatosensory perception or pain responses to stimulation may have affected the firing rates. Since the anesthetic procedure was common to all stimulation conditions, it is unlikely to be the only reason for these results. Second, the shunting effects through the hole used to insert the recording electrode should be considered. Even though dental cement was used as an electrical insulator, it was unclear exactly how much the electrical current may have flowed in this region. However, this is also unlikely to be the only reason for these results, because there are shunting effects in all groups. Third, we did not measure the electrical resistance of the rat. Since tDCS was applied with a constant current and rats received an equivalent amount of current irrespective of the voltage value, this is unlikely to be the only reason for these results. Fourth, there is a significant gap between the present study and clinical settings; the applied current density in the present study was 32.0 A/m^2^, which was higher than that used clinically (e.g., 1–2 mA using 5 × 7-cm electrodes = 0.29–0.57 A/m^2^). In our previous study (Tanaka et al., 2013), which investigated the effect on dopamine release in the striatum, we performed a preliminary investigation concerning the current intensity of stimulation. As a result, we found that 400-μA stimulation loses half of its effect compared with 800-μA stimulation. In consideration of this result, we adopted an 800-μA stimulation for full effectiveness. Furthermore, we used a single-current intensity to minimize any negative impact on the animals. Current density should be considered when tDCS is applied clinically on the basis of the present result. Fifth, since we used a self-assembled preamplifier, impedance is not necessarily high enough to prevent shunting the current. We could not control the accurate current intensity of stimulation under recording; therefore, we are unable to indicate the alteration in neuronal activity during stimulation. Sixth, during spike event isolation using the spike-sorting software EToS, there were some excluded spikes that were not in single units. This may have been a factor for disagreement between results of single-unit and multiunit activities.

In conclusion, we demonstrated that tDCS can induce *in vivo* alterations in neuronal activity in the rat motor cortex beneath the stimulus electrode after stimulation. Our collective findings suggest that the effects of tDCS comprise direct effects on the motor cortex beneath the stimulus electrode and indirect effects in subcortical areas influenced via neuronal networks. As heretofore discussed, many factors influence the effects of tDCS. Therefore, to obtain appropriate effects at the bedside, the contribution of different factors should be identified. Our study lays an important foundation for optimizing the application of tDCS.

## Data Availability Statement

The datasets analyzed in the present study are available from the corresponding author upon reasonable request.

## Ethics Statement

The animal study was reviewed and approved by the Animal Care and Use Committee of the National Institute of Neuroscience (National Centre of Neurology and Psychiatry, Tokyo, Japan).

## Author Contributions

TT, YI, KK, TH, ST, and MH designed the research. TT performed the research and analyzed the data. TT and MH wrote the manuscript.

## Conflict of Interest

The authors declare that the research was conducted in the absence of any commercial or financial relationships that could be construed as a potential conflict of interest.

## Footnotes

1 http://www.R-project.org
